# Low-cost augmented reality goggles enable precision fluorescence-guided cancer surgery

**DOI:** 10.21203/rs.3.rs-3084024/v1

**Published:** 2023-07-12

**Authors:** Leonid Shmuylovich, Christine M. O’Brien, Karen Nwosu, Samuel Achilefu

**Affiliations:** 1 Biophotonics Research Center, Department of Radiology, Washington University in Saint Louis School of Medicine, Saint Louis, MO; 2 Division of Dermatology, Department of Medicine, Washington University in Saint Louis School of Medicine, Saint Louis, MO; 3 Department of Biomedical Engineering, Washington University in Saint Louis, St. Louis, MO, USA; 4 Department of Medicine, Washington University in Saint Louis School of Medicine, St. Louis, MO, USA; 5 Department of Biochemistry & Molecular Biophysics, Washington University, St. Louis, MO, USA

**Keywords:** Fluorescence guided surgery, open source hardware, wearable, augmented reality, Raspberry Pi

## Abstract

Disparities in surgical outcomes often result from subjective than objective decisions dictated by surgical training, experience, and available resources. To improve outcomes, surgeons have adopted advancements in robotics, endoscopy, and intra-operative imaging including fluorescence-guided surgery (FGS), which highlight tumors in real-time without using ionizing radiation. However, like many medical innovations, technical, economic, and logistic challenges have hindered widespread adoption of FGS beyond high-resource centers. To overcome these impediments, we developed the fully-wearable and battery-powered fluorescence imaging augmented reality Raspberry Pi-based goggle system (FAR-Pi). Novel device design ensures distance-independent coalignment between real and augmented FAR-Pi views and offers higher spatial resolution, depth of focus, and fluorescence detection sensitivity than existing bulkier, pricier, and wall-powered technologies. When paired with pan-tumor targeting fluorescent agents such as LS301, FAR-Pi objectively identifies tumors in vivo. As an open-source, affordable, and adaptable system, FAR-Pi is poised to democratize access to FGS and improve health outcomes worldwide.

Surgery is the primary curative method for most localized solid tumors. Surgical outcomes continue to improve for most tumor types due in part to advances in preoperative and intraoperative imaging technologies. However, mortality rates after cancer surgery are disproportionately higher in low than high-resource medical centers^1^, regardless of the aggregate income level of any country. Recent advances in electronics, sensors, and nanotechnology have introduced portable, implantable, and wearable devices capable of reducing surgical outcome disparities. Fluorescence-guided surgery (FGS) is one of such method that enables surgeons to visualize tumors in real-time, identify residual tumors, and assess the extent of disease. Studies have shown that near-infrared (NIR, 700–900 nm) FGS offers high contrast delineation of important anatomy or diseased tissue and at depths exceeding visible light imaging without using ionizing radiation. Ongoing studies suggest that FGS aids in identifying cancer-free margins intraoperatively^2,3^, thus ensuring complete removal of malignant tissue and decreasing the rate of costly re-excision^4^. Reports of early clinical successes and opportunities for broad impact^2,5^ has accelerated the development of new FGS hardware systems^6^ and novel targeted contrast agents for various surgical applications^7,8^. Moreover, FGS’ objective visualize of anatomy and disease supports its potential to level surgical outcomes for patients worldwide. Current FGS hardware designs include standalone, handheld, and wearable systems^6,8^.

Consistent with many medical innovations^9^, the complexity and cost of these FGS systems create barriers to widespread adoption. In previous reports, we attempted to address these challenges by developing a wearable FGS system called Cancer Vision Goggles (CVG), which is a device that is capable of detecting NIR fluorescence of tumor-targeting agents in the operating room^10–12^. While CVG demonstrated the feasibility of this approach in the clinic, hurdles to widespread clinical adoption such as cost, ergonomics, and technical complexity persist. A compact form factor is necessary to fit within space-constrained operating rooms. Further, integrating the device components into a fully wearable unit will eliminate the need for additional support staff to operate the system, which is the case with nearly all FDA-approved FGS systems to date. Further, battery-powered devices will prevent tethering surgeons to cords and allow their use in settings where wall power is unreliable.

Here, we report the development of an inexpensive fluorescence imaging augmented reality Raspberry Pi-based goggle system (FAR-Pi) capable of real-time coaligned visible and NIR background-subtracted imaging. We leveraged open-source hardware and rapid prototyping to incorporate low-cost single board computers (SBC), microcontrollers, and off-the-shelf electronics into the device. We solved a longstanding problem in the field by achieving accurate distance-independent optical alignment between the surgeon’s field of view and the detected visible and NIR image stream. Unlike existing commercial systems, FAR-Pi is fully wearable and battery-powered. By making the design open-source, accessible, inexpensive, and modular, FAR-Pi presents an opportunity to support multiple clinical use cases, foster innovation, allow for local manufacturing in areas where medical exports are limited, and democratize this previously inaccessible technology for the global human health^13,14^.

## Results

**Figure 1.**
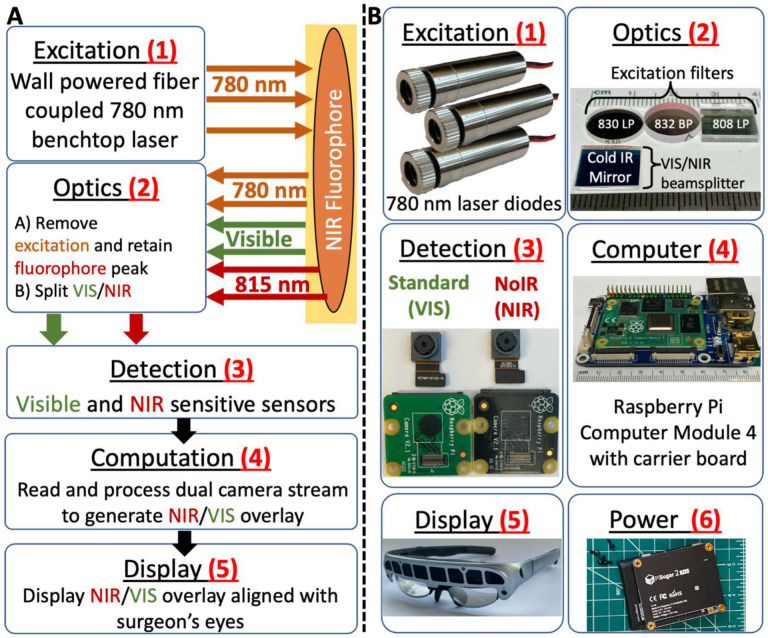
Summary of a representative fluorescence-guided surgery system and opportunities for simplification with an open-source low-cost off-the-shelf redesign. A) Block diagram of a representative see-through display wall-powered non-wearable fluorescence-guided surgical system^10^ which consists of a benchtop 780 nm laser module (1) that is capable of exciting NIR fluorophores, optical elements (2) designed to remove excitation light and split visible and NIR signals, VIS and NIR imaging sensors with control module (3) designed to detect the signal, a laptop computer (4) that processes image sensor data to generate a false color NIR image, and an optical see-through display (5) that accepts input from the computer and provides the user with an NIR fluorescent signal superimposed over their field of view. The entire system is powered through wall power. B) To create a simpler, low-cost, and more accessible fluorescence-guided surgical system, the feasibility of replacing each existing component with less expensive off-the-shelf items was evaluated. Proposed alternatives include (1, Excitation): replacing the existing benchtop fiber-coupled laser module with an array of inexpensive 780 nm laser diodes, (2, Optics): using inexpensive cold-infrared mirror as a beamsplitter and a variety of potential excitation filters, (3, Detection): detecting both visible and NIR signals using a Raspberry Pi v2 cameras that detect visible only (left sensor) and visible plus NIR light (right sensor), (4, Computation): processing and outputting imaging data using a Raspberry Pi (bottom) single-board computer, and (6, Power): powering the entire system using a rechargeable Raspberry Pi uninterrupted power supply.

NIR FGS systems generally consist of: 1) NIR excitation light, 2) optical filters to distinguish visible from NIR light and remove excitation light, 3) visible and NIR camera sensors, 4) a computational device that controls system components, processes the detected camera data to ensure clinical utility, and outputs the processed data, 5) a mechanism for displaying meaningful clinical output to users, and 6) a power source (Figure 1A)^6,10^. To create an open-source FGS system that overcomes existing FGS challenges while also decreasing clinical footprint, complexity, and cost^6^, we leverage the tools of the ‘Maker Movement’^15^ to redesign each component (Figure 1B).

### Laser diode array as an excitation light source

**Figure 2.**
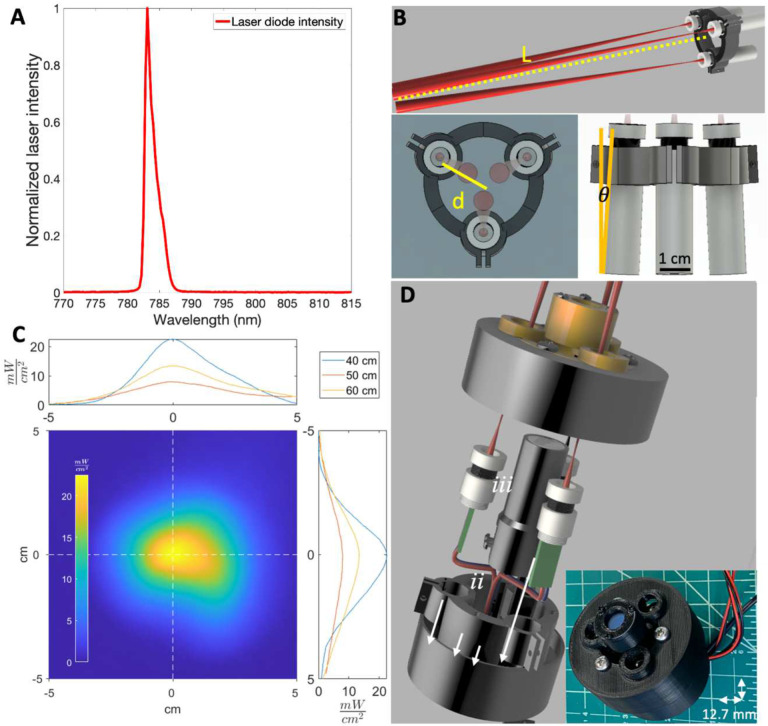
The FAR-Pi illumination module, consisting of a multi-purpose circular 780 nm laser diode array, delivers sufficient power for the FAR-Pi imaging module to operate at a working distance of 50 cm. A) Spectral characterization of a Laserlands 120 mW 780 nm laser diode demonstrating peak intensity at 783 nm with <2 nm full-width at half maximum. B) Parametric CAD model demonstrating a circular arrangement of laser diodes. The working distance *L* between laser array and the point where individual lasers intersect on the surgical field, and distance *d* between individual laser center and array center, determine the angle of laser inclination *θ*. C) Laser diode array spatial power distribution was measured at working distances of 40 cm, 50 cm, and 60 cm. The spatial power distribution at 50 cm is shown in the bottom left panel as a 2 dimensional heatmap, and horizontal and vertical cross sections at 40, 50, and 60 cm are plotted in the top and right panels. At working distances of 50 cm, laser power exceeded 5 mW/cm^2^ over a 5.4 cm diameter area. D) CAD model (left) and physical component build (right) of a 3D-printed implementation of the illumination module where the FAR-Pi imaging module is positioned in the center of the laser diode array. E) CAD model with visible internal components and physical build of a 3D-printed implementation of the illumination module with central white light LED (*ii*), surrounding laser diode (*i*) array, and front-facing optical filter assembly (*iii*).

We first evaluated whether an array of handheld inexpensive battery-powered 120 mW 780 nm laser diode modules could provide an alternative to existing bulky, expensive, and wall-powered FGS excitation sources^6,10,16–18^. The laser diode demonstrated a peak intensity at 783 nm with full width at half maximum of 1.5 nm (Figure 2A). A compact battery powered circular array of diodes (Figure 2B) had a maximum power between 20 and 5 mW/cm^2^ at distances of 40 to 60 cm (Figure 2C). At a distance of 50 cm, the irradiated power exceeded 5 mW/cm^2^ over a 5.4 cm diameter area, which is within the 5–10 mW/cm^2^ range utilized in existing FGS systems^6^. Combining the circular diode array with a central battery-powered white light LED and shortpass IR filter (Figure 2D) resulted in a 110-gram assembly with cylindrical dimensions of 64 mm diameter base, 60 mm height.

### NIR and visible light detection with coaligned RPiV2 cameras

We next evaluated whether RPiV2 cameras could achieve dual NIR and visible imaging similar to some existing FGS systems. RPiV2 camera spatial resolution ranged from 138–227 μm at distances between 35–65 cm. This aligns with theoretical predictions (Supplement, Section 1, Figure S2) and is within the 50–500 μm spatial resolution range of existing FGS systems. However, the 20 cm depth of field far exceeds the 2–3 cm depth of field of existing FGS systems^6,19^.

**Figure 3.**
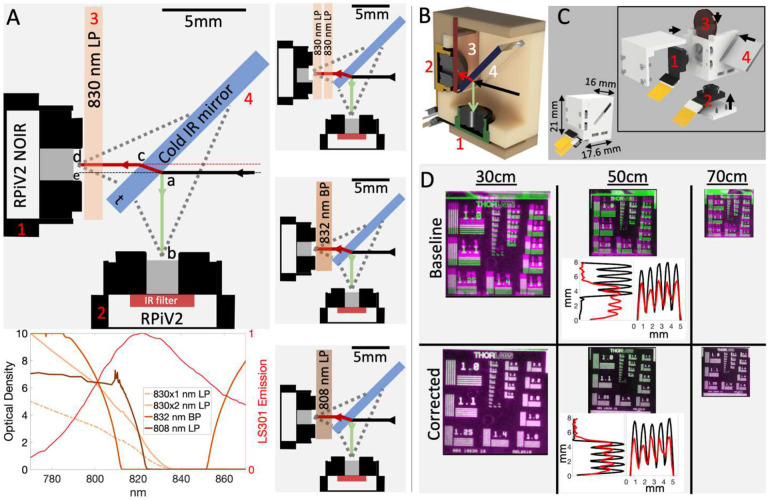
Raspberry PiV2 camera sensors can be combined with inexpensive optical components in a compact 3D-printed enclosure to achieve coaligned dual visible and NIR imaging. A) To-scale schematic demonstrating optically aligned cameras (items 1,2), optical components (items 3,4), and optical paths of visible (green line, path *ab*) and NIR light (red line, path *acd*). Majority of incident visible light is reflected off the cold IR mirror into an RPiV2 camera sensor (item 2) with internal IR filter. Majority of incident NIR light is refracted through the cold IR mirror, and then passed through an excitation light filter before entering an RPiV2 camera NoIR sensor (item 1). Four excitation filter options are outlined, and in the lower panel the optical density each filter combination plotted and compared with the normalized emission spectrum of LS301, a tumor-targeting NIR fluorophore^22^. B-C) Cutaway and assembled view of all components positioned within a 3D printed enclosure. D) Superimposed visible and NIR images of a resolution test target captured at multiple distances demonstrates distance-dependent alignment error at baseline. The middle panel shows pixel intensity along resolution target horizontal and vertical line pairs from group 1.0 at 50 cm. Red peaks correspond to the visible image line pairs, black peaks correspond to NIR image line pairs. The degree of overlap between red and black peaks is a measure of coalignment between visible and NIR images in horizontal and vertical axes. An affine transform defined from corresponding fiducial points obtained from the 50 cm NIR and visible images achieves near-perfect alignment between NIR and visible camera images independent of distance.

Alignment of the RPiV2 NoIR and standard camera sensors with a cold IR mirror (Figure 3A–C) in a 3D-printed enclosure resulted in a lightweight (< 5g) and compact (<6 cm^3^) imaging module with an alignment error between NIR and visible images of 1 pixel horizontally and 20 pixels vertically. This error translates to 3 mm vertical deviation at a 50 cm imaging distance (Figure 3D, Baseline). A projective transform determined by calibration at a single distance (50 cm) ensured alignment within 1 pixel between visible and NIR images at any distance (Figure 3D, Corrected).

### Computation module

Raspberry Pi SBCs are less computationally powerful than computers used in existing FGS systems^6,10,17,19^, but have the advantage of being wearable and relatively inexpensive^20^. We therefore tested if Raspberry Pi SBCs had sufficient computational power to support FGS.

#### Dual-camera Streaming

We achieved dual-camera streaming with both the Compute Module 4 (RPiCM4) and the Raspberry Pi V4 SBC (RPiV4). With the use of Python threaded processes, the PiCamera package, and OpenCV methods, the RPiCM4 could read, process, modify, and display images via HDMI port in real-time. CSI to HDMI conversion boards, which allow the video signal to be transferred via HDMI cables, ensured video signal fidelity over 5 meters (Supplement, Sections 3A-C, 5A).

#### Laser diode array control and synchronization

**Figure 4.**
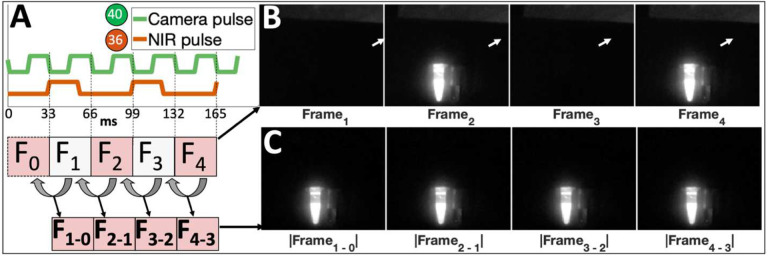
Synchronization of laser excitation with alternating camera frames facilitates real-time NIR-background subtraction. A) The device tree of the Raspberry Pi computer is modified to toggle GPIO pin 40 high/low with camera frame read start/stop events, generating a square wave (camera pulse, green) with 33 ms period when imaging at 30 frames per second. B) Triggering GPIO pin 36 (which toggles the entire laser diode array) at the end of every other frame (double the period of the camera pulse) toggles laser excitation with each frame, resulting in detected fluorescence from an Eppendorf tube filled with indocyanine green (ICG) on frames 2 and 4 in a sequence of 4 consecutive frames acquired with a NoIR RPiV2 camera fitted with a Newport 830 nm longpass filter. Note background NIR signal from room lights reflected off white paper near top of each acquired frame (white arrow). C) The absolute value of the difference between the current frame and the preceding frame generates a real-time video stream preserving the emitted fluorescence signal and removing the background NIR signal.

To synchronize frame capture and laser excitation, we developed a custom ‘HAT’ (Hardware Attached on Top) circuit board that provides software-based laser control via Raspberry Pi GPIO pins (Figures S8, S9). Triggering the laser diodes to turn on with every other frame (every 66 ms) and turn off after 70% of the duration of a frame (Figure 4A) resulted in an image stream where NIR-fluorescence from an indocyanine green (ICG) sample toggled with consecutive frames (Figure 4B). Longer laser excitation duration resulted in bleed-through of laser excitation on consecutive frames. The absolute value of subtracted consecutive frames (ΔF_n_ = |F_n_ − F_n-1_|) yielded a continuous 30 frames per second (fps) NIR-background-subtracted stream (Figure 4B–C).

#### FAR-Pi NIR Fluorescence Sensitivity

**Figure 5.**
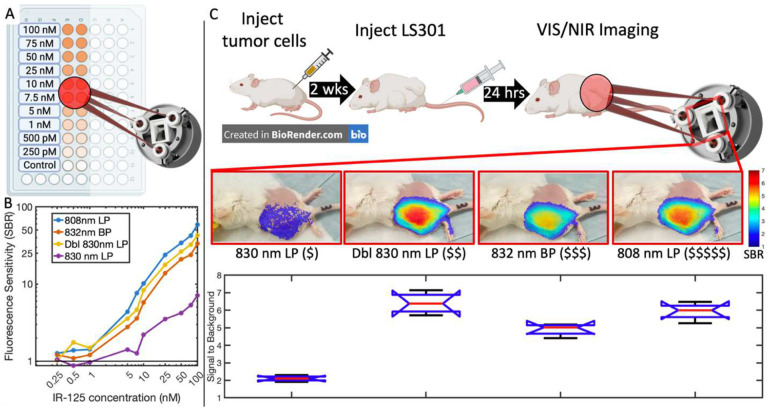
FAR-Pi is sensitive to 1nM fluorophore concentrations and can detect cancerous tissue in an in vivo mouse model. A) Schematic of experimental determination of FAR-Pi fluorescence sensitivity limits using a 96 well plate with IR-125 concentrations ranging from 250 pM to 100 nM. The difference between images obtained with laser on and laser off defined an NIR-background-subtracted image for each well. Fluorescence signal to background ratio (SBR) was calculated for each well by dividing the average background-subtracted pixel intensity of a given well by the average detected background-subtracted pixel intensity in the control well. B) A log-log plot of SBR vs IR-125 concentration for 4 different excitation filter options outlined in Figure 3A: a single 830 nm Newport longpass filter (LP), two 830 nm LP filters in series (Dbl 830 nm LP), one 832 nm Edmund bandpass filter (BP), or one 808 nm Semrock LP filter. C) In vivo validation in a mouse model with left flank subcutaneous breast cancer tumor demonstrating NIR fluorescence from the cancer-targeting NIR fluorophore LS301 measured with each of the FAR-Pi imaging module excitation filter options. The $ signs next to each filter name reflect relative expense. In each case coaligned simultaneous visible and NIR images captured with laser on and off, the NIR signal is processed to generate an NIR-background subtracted image, and then the background subtracted image is divided by an excitation filter-specific background intensity to generate an LS301 SBR heatmap. The LS301 SBR heatmap is shown as a partially transparent overlay on the coaligned visible image using a threshold of SBR>1.5. Lower panel is a box and whisker plot quantifying the peak LS301 SBR value across all mice grouped by the type of excitation filter used during data acquisition. Median is shown in red, top and bottom lines show 75^th^ and 25^th^ percentile respectively. The blue notch indicates a 95% confidence interval around the median.

A stable ICG-matching phantom consisting of durable polyurethane with varying concentrations of IR-125 dye^21^ (Figure 5A) was used to characterize the fluorescence signal-to-background ratio (SBR) of the FAR-Pi system with four alternative excitation filter designs (Figure 5B). The least expensive single 830 nm longpass (LP) filter had the lowest SBR and was particularly poor below 10 nM, indicating significantly more leakage of reflected excitation light than alternative filters. The other excitation filter options showed a linear log-log relationship between SBR and IR-125 concentrations from 100–1 nM, followed by a flat plateau. An inexpensive double 830 nm LP design showed similar performance to the more expensive options.

#### In vivo evaluation

The ability of the FAR-Pi system to detect in vivo accumulation of an intravenously administered cancer-targeting fluorophore^22^ was tested using a subcutaneous breast cancer model in *n*=4 mice. Figure 5C shows a coaligned overlay of the visible camera image with the NIR-camera-derived LS301 SBR spatial map for four different excitation filter options in one mouse. Across all mice the average LS301 peak SBR signal (Figure 5C, lower panel) measured with the single 830 nm LP filter was significantly lower (2.11±0.16) than LS301 peak SBR measured with the double 830 nm LP filter (6.40±0.62, *p* = 5.5•10^−8^), the 832 nm BP filter (4.9±0.35, *p* = 6.0•10^−6^), or the 808 nm LP filter (5.93±0.50 *p* = 2.0•10^−7^). LS301 peak SBR was significantly higher when measured with the double 830 nm LP than with the 832 nm LP filter (*p* = 0.0021) but not significantly different (*p* = 0.47) when compared to the 808 nm LP filter. Finally, LS301 peak SBR was significantly higher when measured with the 808 nm LP than with the 832 nm BP filter (*p* = 0.029).

### Alignment of visible and NIR cameras with user’s view

**Figure 6.**
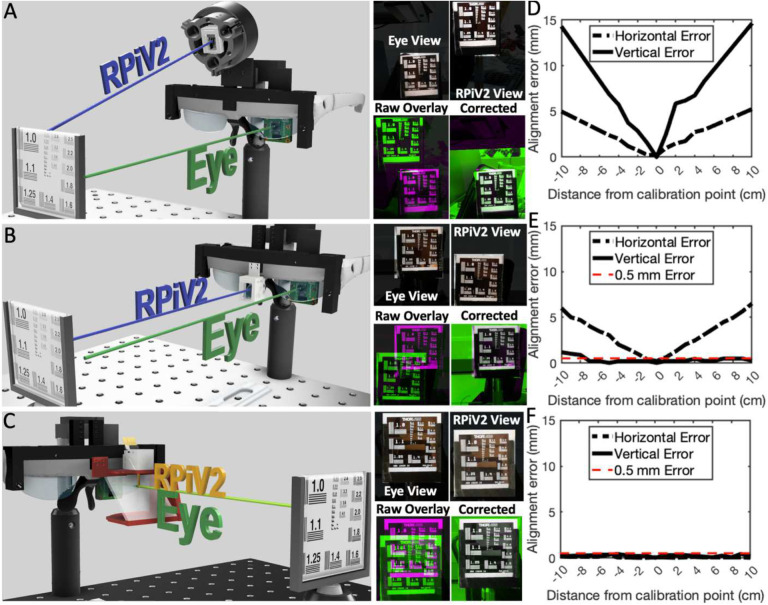
Alignment between FAR-Pi imaging module camera and surgeon’s view can be assured through software-based projective transformation at fixed distance, and can be assured at any distance through optical alignment of camera and eye. A) Experimental setup to test the alignment error of resolution test target images obtained by a surrogate eye camera (‘eye view’) and an imaging module (‘RPiV2 View’) positioned within a laser array that is mounted to heads up display (HUD) glasses coupled to custom 3D-printed mounting assembly. Image overlay (‘Raw Overlay’) highlights image misalignment. A 4-point projective transform defined by corresponding fiducial points between images corrects this misalignment and provides an error-free image overlay (‘Corrected’). B) Repeating the analysis of A) with an alternate design, where the imaging module is placed at eye level between the eyes. C) Repeating the analysis of A) with another design, where a glass beamsplitter is placed in front of the HUD glasses oriented 45 degrees relative to the eye light path, and the imaging module is positioned above the beamsplitter in line with the reflected light path. In this arrangement the light paths for imaging module camera and eye surrogate camera are coaxial. D-F) Horizontal and vertical alignment error between the transform-corrected imaging module camera image and eye surrogate camera image as the distance between eye surrogate camera and resolution target varies from 40 cm to 60 cm (±10 cm from the calibration distance). The results in D, E, and F correspond to the experimental setups in A, B, and C respectively.

In a see-through FGS system, misalignment between the projected fluorescence signal and the surgeon’s view could result in misinterpretation of tumor position and inaccurate tissue excision. We therefore measured misalignment between the camera and surgeon’s view by placing a resolution target 50 cm from a camera that mimics the surgeon’s eye, and then positioned the FAR-Pi imaging module either at (1) mid-forehead, (2) centered at eye level, or (3) optically coaligned with the eye using a beamsplitter (Figure 6A–C).

Misalignment was most significant (69.5 mm vertical and 26.5 mm horizontal offset) at position (1) (Figure 6A), less significant (36.9 mm vertical and 23.0 mm horizontal offset) at position (2) (Figure 6A,B), and minimized in position (3), where the eye and imaging module are optically coaxial (17.3 mm vertical and 3.4 mm horizontal offset) (Figure 6C). In each case, a four-point projective transformation derived from matching fiducial markers on resolution target images captured at a 50 cm calibration distance corrected misalignment (Figure 6A, B, C ‘Corrected’). However, with the target moving 10 cm closer or further from the calibration distance, vertical and horizontal alignment errors increased to 15 mm and 5 mm, respectively, for position (1) and increased to 1 mm and 5 mm, respectively, for position (2) (Figure 6D–E). In contrast, in position (3) total alignment error remaining less than 0.5 mm vertically and 0.3 mm horizontally as the target moved 10 cm closer or farther from the calibration distance (Figure 6F). Given that the beamsplitter-based design (Figure 6C), where the eye and imaging module are optically coaxial, ensures distance-independent coalignment between the FAR-Pi image module and the surgeon’s eye, this design was chosen for the full FAR-Pi implementation.

### Combining all components into a fully wearable system

**Figure 7.**
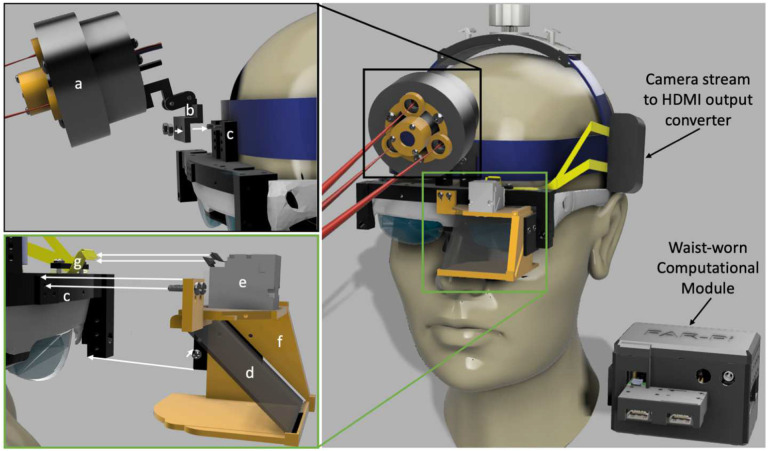
Integrating illumination and imaging modules made from 3D printed and off-the-shelf components with a heads up display and Raspberry Pi computer results in a fully wearable and battery-powered fluorescence-guided surgery (FGS) system. CAD rendering of wearable FAR-Pi FGS system with illumination, imaging, and Rokid Air augmented reality (AR) glasses mounted to surgical headgear and waist-mounted computational module. Left upper panel shows how the illumination module (*a*), detailed in Figure 2E, is secured to an articulating arm (*b*) with is then secured to center mounting holes (*c*) on a custom component coupled to the AR glasses. Left lower panel shows a custom component (*f*) that holds the imaging module (*e*) and a 45° beamsplitter (*d*). Securing this component to the AR glasses coupling component (*c*) makes the eye and imaging module optically coaxial. Flexible MIPI cables (*g*) are clamped to the AR glasses and connect to the two imaging module RPiV2 camera sensors secured within the imaging module enclosure. Right upper panel shows the components of the head-mounted enclosure (*j*) responsible for video HDMI conversion. The MIPI cables (*g*) from the RPiV2 camera sensors connect to HDMI-CSI converter boards which convert the RPiV2 camera signal into an HDMI video signal that can be extended over large distances. HDMI cables (not shown) carrying the dual camera stream are connected to camera input ports on a waist-worn battery powered computational module enclosure. Wires providing power and control of the illumination module also connect to the computational module enclosure (wires not shown).

**Figure 8.**
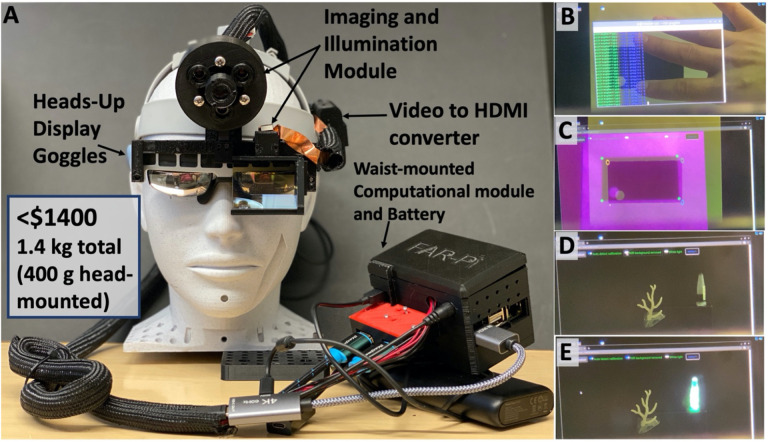
FAR-Pi system demonstrates 10x improvements in cost and weight compared to prior systems. **A)** Full redesigned FAR-Pi system worn on a mannequin head. The computational module and Anker battery pack are waist-mounted, and the remaining components are integrated into a wearable head mounted assembly. The head mounted components weigh 400g, and the waist-mounted components add an additional 1 kg. The FAR-Pi implementation shown here includes Rokid Air augmented reality glasses, a beamsplitter splitter assembly to make the imaging module coaxial with the surgeon’s eye, an illumination module with central white light, a video HDMI conversion kit, and multiple 3D printed components. The total cost of this implementation is less than $1400. Unlike previous systems that are orders of magnitude more expensive and bulky, the FAR-Pi does not require wall power, is fully wearable, and does not tether the user to a cart. B) Placing an iPhone camera behind the Rokid Air glasses provides a visualization of what the surgeon sees while wearing the FAR-Pi system. In B) the surgeon sees their hand and a calibration target in the real world, with the Raspberry Pi terminal window superimposed in their field of view. C) After launching the FAR-Pi software application, an initial calibration step ensures that the camera signal from the FAR-Pi system (purple overlay with green closed circles) coaligns with the surgeon’s view (darker black square). The system automatically detects calibration target corners (green circles), and after the surgeon selects corresponding corners in their field of view (open circles), a transformation is defined that provides real-time coalignment between the camera signal and the surgeon’s view. D) Both a vial of 1mM ICG and the FAR-Pi software application window is visible in the surgeon’s field of view, note there is no NIR signal overlay from the ICG with the laser turned off. E) Once the laser pulse (synchronized with camera frame rate) is turned on, the background-subtracted NIR fluorescence signal detected from the ICG vial is transformed in real-time based on the calibration step in C) and superimposed in false color green in the surgeon’s field of view.

The FAR-Pi illumination, imaging, and computational modules were combined with customized (Figure S15) off-the-shelf HUD see-thru augmented reality glasses (Rokid Air, Rokid, Hangzhou, China) into a single compact and light fully wearable system (Figure 7, Figure 8A) with computational module worn on the waist and remaining elements head-mounted. Figure 8B is a view through the glasses looking at a hand (captured by placing an iPhone behind the glasses) where the Raspberry Pi terminal window is seen superimposed over the real-world view. To ensure distance-independent alignment between FAR-Pi cameras and surgeon’s eye, the user calibrates the system at startup by selecting points in their real-world view that correspond to automatically detected edges of a rectangular calibration target (Figure 8C). Once fluorescent signal is detected, the surgeon’s view shifts from having no overlay (Figure 8D) to having an overlay with the FAR-Pi detected NIR signal coaligned with their real-world view (Figure 8E). The head mounted components had a total weight of 390 g, and the waist-worn computational module had a total weight of 580 g. A 5000 mAh PiSugar power supply coupled to the RPiCM4 powered the FAR-Pi system for 1 hour. Adding a 460 g 20,100 mAh 5V 4.8A portable USB Anker PowerCore power bank to the waist-worn assembly extended operation by four additional hours.

## Discussion

FGS is a powerful clinical tool with the potential to have a profound impact on clinical outcomes, but its cost and complexity make it inaccessible to most patients around the world. While our lab and others^10,12,17–19,23–27^ have introduced innovations to transform FGS from a tethered cart-based system where a surgeon views the fluorescence signal on an external monitor to a wearable head-mounted system, head-mounted see-through FGS systems continue to be wall-powered, complex, expensive, relatively bulky, and replete with significant mismatch between the detected NIR signal and the surgeon’s view. In this work, we show that inexpensive off-the-shelf components coupled with tools of the “Maker Movement”^15^ can be used to create a novel battery-powered FGS system with accurate alignment between detected NIR and surgeon’s view where all components (illumination module, imaging module, computation module, and heads-up display) are compact, accessible, and wearable.

Existing FGS systems employ large and expensive cart-based laser diodes, effectively tethering the surgeon to the cart with optical fibers^6^. We found that a low-cost, compact, fully-wearable, and battery-powered laser diode array provides similar irradiance to tethered systems while freeing the surgeon from being connected with cables to a cart or wall power. Some groups have utilized less bulky LED modules, but these have broad spectra and, unlike the laser diode array, require additional clean-up filters to ensure that their NIR tails do not overlap with the emitted fluorescence signal^17^. A halogen lamp^18^ is a relatively inexpensive alternative, but it also requires additional optical filters and poses a challenge for wearable devices given that it is wall-powered and generates significant heat.

Though commercial FGS systems rely upon more expensive camera sensors (i.e. CCD, EMCCD, ICCD, or sCMOS) with ≥10 bit-depth and enhanced NIR quantum efficiency^6^, we found that a low-cost RPiV2 NoIR CMOS camera sensor operating at 8-bit depth with real-time exposure times (< 40 ms) could detect as low as 1–10 nM concentrations of IR-125 dye, thereby rivaling the performance of several commercial FGS systems. Kim et al also showed that the RPiV2 NoIR camera can detect NIR fluorescence in a medical application^28^, but neither fluorescence detection limits nor laser power was characterized, and signal detection required 2-second exposure times (not compatible with real-time imaging). The use of flexible MIPI connection cables allowed the RPiV2 NoIR imaging sensor and lens housing to be closely positioned with a beamsplitter and a second standard RPiV2 sensor (sensitive to visible light only) in a compact (~6 cm^3^) 3D-printed enclosure. We found that a software-based projective transform, determined from a calibration step at one distance, could be applied at any distance in real-time to achieve optically coaligned dual white light and NIR imaging. This represents an innovative approach where software correction is able to overcome inaccuracies inherent in aligning components using 3D printed parts and underscores the potential for low-cost manufacturing practices like 3D printing to support the development of high-quality medical technology in low-resource settings.

Other groups have shown how a custom single camera sensor can achieve optically coaligned dual white light and NIR imaging^24,25^. However, resolution suffers with multispectral sensors because only a subset of camera pixels is reserved for a particular wavelength. In addition, multispectral cameras are not easily accessible – at the time of writing, no off-the-shelf RGB-IR cameras (such as See3CAM-CU40) were commercially available.

A Raspberry Pi SBC has been used in a portable projection-based NIR FGS system by Li et al., but that system lacked a second visible camera stream, relied on long exposure times (330 ms), and used a camera that was an order of magnitude more expensive than the RPiV2^20^. In this work we show how a Raspberry Pi SBC can be combined with RPiV2 cameras and a compact laser array to create an FGS system that is battery-powered, wearable, and capable of dual visible/NIR imaging with real-time NIR-background subtraction through pulsed synchronized laser excitation. While real-time NIR-background subtraction through synchronized laser pulsing has been described^27^, it is not implemented^6^ in many commercial FGS systems (requiring operating room lights to be dimmed for those systems), and to our knowledge, it has previously not been implemented with Raspberry Pi SBCs and RPiV2 cameras.

Several innovative immersive (not see-through) virtual reality (VR) dual visible and NIR FGS HMD systems have been proposed^17–19,26,27^. While VR is well suited for endoscopy and laparoscopy, an augmented reality HMD system where the surgeon can still see the surgical field in open surgery is preferred. Ensuring alignment between what is displayed on the see-through HMD and what the surgeon sees with their eyes is crucial – without proper visual alignment the surgeon may excise benign tissue while leaving malignant tissue behind. In this work we show that when the camera and eye are not optically aligned (as is typically the case in existing see-through systems^10,29–31^), alignment can be achieved by calibrating at a fixed distance, but this corrective measure fails at distances beyond the calibration distance. To overcome this limitation and ensure that our system accurately aligns the detected NIR and visible camera signals with the surgeon’s eye at any distance, we introduced a novel beamsplitter approach that makes the camera optically coaxial with the surgeon’s eye. This dramatically simplifies the FGS system by removing the need for additional hardware and complex software associated with distance-specific camera signal transformation. A similar approach was introduced by Lee et al., but unlike our system, their work was limited by not having a coaligned visible camera feed^32^.

While a custom-built AR HMD would provide more engineering flexibility, our goal was to make the FAR-Pi simple to source and build, and therefore we repurposed off-the-shelf AR glasses (the Rokid Air) for medical imaging. Off-the-shelf augmented reality glasses can be challenging to utilize when feeding a non-stereoscopic camera feed into them because they typically mirror the same image to both the left and right eye, creating a sensation of double vision. We overcame this limitation by modifying the Rokid Air to display the monocular signal to only one eye (Figure S15). We are not aware of a similar modification working with other off-the-shelf augmented reality glasses, and previously published non-stereoscopic FGS systems do not address methods for overcoming this double vision limitation^32^.

There are several limitations that can be improved upon in the FAR-Pi system. The FAR-Pi laser diodes have a weak but long NIR tail that contributes to a weak but non-zero background signal that decreases SBR. While the effect is negligible at fluorophore concentrations exceeding 1 nM, FAR-Pi system sensitivity to sub-nM fluorophore concentrations could be improved by adding excitation clean-up filters (see Figure S5, S7). The RPiV2 cameras use fixed focus lenses, and while the depth of focus of the RPiV2 lens ensures appropriate focus for the likely range of distances encountered in practice, implementing motorized focus would increase the range of working distances supported by the FAR-Pi system. The illumination module consists of 3 lasers at fixed angle of inclination, with a Gaussian power distribution at the excitation surface. Dynamically adjusting the lasers’ angle of inclination could ensure a more homogenous power distribution at a given working distance. The FAR-Pi system uses a single NIR camera and therefore does not capture depth information. A stereoscopic dual visible/NIR imaging system would support stereoscopic display.

In summary, the FAR-Pi system is an open-hardware inspired, fully wearable, head-mounted, battery-powered, and relatively easy to build augmented reality FGS solution that is an order of magnitude smaller, lighter and less expensive than existing systems. Incorporating features that allow optical alignment of the imaging module cameras with the surgeon’s view uniquely improves the reliability and capability of the FAR-Pi system, bringing the goal of globally accessible FGS within reach. Further development of the FAR-Pi system is poised to improve human health by making the benefits of FGS available to patients around the world.

## Methods

### Laser Diode Array Characterization

The normalized spectral profile of ~$20 120 mW 780 nm laser diodes (Laserlands.net , Besram Technology Inc, Wuhan, China)) was measured with a visible-NIR spectrometer (USB2000+VIS-NIR-ES, Ocean Insight, Orlando, FL) (Figure 2A). A laser array consisting of three 780 nm laser diodes that intersect at 40 cm and have space for a central imaging module (Figures S5, S6) or white light LED (Figures 2D, S7) was constructed and aimed at a black posterboard 40–60 cm away. An optical power meter (S121C and PM100D, Thorlabs Inc, Newton, NJ) was used to determine maximum power on the posterboard. Surface temperature (T) of the posterboard was measured with a FLIR T650Sc thermal camera (FLIR Systems, Wilsonville, OR). Surface temperature maps before (T(x,y)_*t*=0_) and after laser illumination (T(x,y)_*t*=5_) were extracted using the FLIR ATLAS MATLAB SDK^33^. Subtracting T(x,y)_*t*=0_ from T(x,y)_*t*=5_ yields ΔT(x,y). Multiplying ΔT(x,y)/DT_MAX_ by measured maximum laser power provided an estimate of the surface laser power distribution (Figure 2C).

#### Dual Visible and NIR imaging

The 8 MP RPiV2 camera was chosen for the FAR-Pi imaging module because it comes in visible and NIR versions (standard and NoIR respectively). Furthermore, miniaturizationis facilitated by detaching the RPiV2 camera sensors from the RPiV2 PCB and connecting with flexible extension cables (B0186, Arducam, Nanjing China) (Figure S1). A 3D-printed enclosure was designed to position a 45° cold IR mirror plate beamsplitter (#62–634 Edmund Optics, Barrington, NJ) between the RPiV2 cameras so that visible light is reflected to the standard camera and optically coaligned NIR light is transmitted to the NoIR camera (Figure 3A–C). To remove excitation light that may overlap with fluorophore emission, multiple filters (placed in front of the NoIR camera) were evaluated: 1) an OD 3–4 12.7mm 830 nm LP Newport filter ($40, 5CGA-830, Newport, Irvine CA), 2) two stacked 12.7 mm 830 nm LP Newport filters, 3) a 12.5 mm OD6 Edmund 832 nm BP filter ($240, #84–091, Edmund Optics, Barrington, NJ), and 4) a 12.5 mm OD6 808 nm Semrock EdgeBasic LP filter ($535, BLP01–808R-12.5-D, AVR Optics, Fairport, NY). OD vs wavelength for each filter is summarized in Figure 3A. For each optical filter, the positions of the cameras and beamsplitter required minor modificationsto ensure coaligned and equal optical path (Figure S3, S4).

Camera coalignment was measured by positioning dual-camera assemblies 30–70 cm from a back-illuminated resolution target, capturing images from both cameras, and then calculating the horizontal and vertical offset between corresponding fiducial markers (Figure 3D, baseline). Corresponding fiducial marker positions were then utilized to derive distance-independent corrective projective transforms between the visible and NIR images. Additional details about the RPiV2 cameras, imaging module enclosures, and camera alignment process is provided in Supplement Section 1.

### Computational Module

To achieve dual-camera streaming we utilized a $65 CM4104016 Raspberry Pi Compute Module 4 (RPiCM4) with a WaveShare carrier board that contains two camera serial interface (CSI) inputs ($20, CM4-IO-Base-A, Waveshare, Shenzhen, China). A custom laser diode control circuit, PiSugar2 Plus 5000 mAh power supply (PiSugar, Guangzhou, China), cooling fan, and camera related hardware were combined with the RPiCM4 in a 3D printed enclosure (Figure 7, S12). Assembly details including alternative approaches to achieve dual-camera streaming and to make the computational module head-mounted are provided in Supplement Section 4 (Figures S8, S10, S11, S13, and S14).

We modified the RPiCM4 device tree to allow synchronization of laser excitation with every other camera frame, and then subtracted consecutive frame pixel-by-pixel grayscale intensities to generate a real-time NIR-background subtracted image stream (See Supplementary Section 3D for additional details). Synchronized excitation was tested by imaging two Eppendorf tubes filled with 1mM indocyanine green (ICG) and water (negative control) respectively. The lack of signal from a control vial of water next to the ICG vial confirmed that reflection of excitation light was not responsible for the detected signal.

#### Software support for dual-camera streaming and display

Python scripts were developed to read and process the dual-camera stream. A Flask web application was written to process and deliver camera signals both wirelessly to a separate monitor as well as directly to an HDMI-input HUD. With the Flask app, the RPiCM4 functions as server, and accessing specific routes on the server through a web-browser generates user-specific application views. When viewed by the surgeon, the app provides control over illumination, performs calibration between FAR-PI cameras and the surgeon’s eyes, and displays the aligned NIR video stream (Figure 8B–E, S19). On an alternate view the app provides both the NIR and visible video streams wirelessly to a remote viewer (Figure S20). Additional software-related details are provided in Section 5 of the Supplement.

### NIR Fluorescence Sensitivity

To determine NIR sensitivity limits of the RPiV2 NoiR camera, a durable NIR fluorescence phantom was made, consisting of a black 96-well plate (Corning, Corning, NY) with duplicate wells of polyurethane embedded with IR-125 dye in concentrations of 0 (control), 250 pM, 500 pM, 1 nM, 5 nM, 7.5 nM, 10 nM, 25 nM, 50 nM, 75 nM, and 100 nM, as described by Ruiz et al^21^ (Figure 5A, Supplementary Section 6).

FAR-Pi imaging modules with 4 separate excitation filter options were placed in a fixed position 45 cm above the IR-125 phantom. The phantom was placed on custom 2-axis motorized stage, which allowed automated positioning of the phantom to ensure consistent 20 mW/cm^2^ irradiation when imaging a given well. At each well, duplicate RPiV2 visible and NIR images were obtained with laser ON and OFF, at multiple exposure times (33 ms, 66 ms, 99ms), with and without auto white balance (AWB), and with ISO set to 400, 600, and 800. A python script automated stage positioning and data acquisition. Signal to background ratio (SBR) for each IR-125 dye concentration was determined in MATLAB by dividing background-subtracted pixel intensities in each fluorophore-containing well by background-subtracted intensities in the control well.

### In vivo studies

All animal studies were performed under an approved protocol by Washington University in St. Louis’s Institutional Animal Care and Use Committee. Animals were housed under a 12 h dark-light cycle. Adult Fox Chase SCID Beige mice (*n* = 4) were subcutaneously implanted with 5•10^5^ 4T1 breast cancer cells on the left dorsal flank. After 2 weeks tumor growth was evident and then tail vein injection of 100 μL of 60 μM LS301-HSA (an NIR cancer-targeting fluorophore developed in our lab^22^) was performed. After 24 hours, mice were imaged (at a distance of 45 cm) using the FAR-Pi system (with 4 different excitation filter options). Coaligned visible and transcutaneous fluorescent NIR images were obtained with laser ON and OFF with 1920×1080 resolution and 33 ms exposure. For the NIR camera ISO was 800 and AWB was OFF. For each mouse an NIR-background-subtracted image was generated by subtracting grayscale pixel intensity of the laser-OFF image from the laser-ON images. While this process removes background NIR room light, leaked NIR light from imperfect excitation filters remains. To determine the degree of leaked signal through each excitation filter, FAR-Pi NIR images of white paper were obtained with laser ON and OFF. The maximum pixel intensity (out of 1) of laser-ON minus laser-OFF images of white paper for the 830 nm LP filter assembly was 0.53. The double 830 nm LP assembly, 832 nm BP filter, and 808 nm LP filters were more effective excitation light filters with maximum pixel intensity of leaked excitation light of 0.082, 0.106, and 0.090 respectively. These pixel intensities set an excitation-filter-specific threshold to which pixel intensities were normalized to generate a pixel by pixel signal to background (SBR), where SBR of 1 corresponds to an NIR signal that is indistinguishable from leaked excitation light. The maximum value of LS301 SBR across all pixels defined the peak LS301 SBR for each mouse under each excitation filter option. In order to exclude signal from non-specific LS301 binding, a mask that excluded pixels with SBR less than 1.5 was applied, and then the image was overlaid with partial transparency over the coaligned visible FAR-Pi image (Figure 5C middle).

### FAR-Pi image alignment with surgeon’s view

The durability of software-based realignment between the FAR-Pi camera and surgeon’s eye was evaluated for 3 designs shown in Figure 6A–C (see Figure S17, S18 for construction details). In the third design the FAR-Pi imaging module and eye are made optically coaxial by placing a 45° beamsplitter (#68–430, Edmund Optics) in front of the HUD glasses, and placing the FAR-Pi imaging module above the beamsplitter (Figure 6C).

An RPiV2 camera was positioned behind the left eye of the HUD to serve as a surrogate for the surgeon’s eye, and then images of a resolution target placed at 1 cm intervals 40–60 cm away from the HUD were captured from the FAR-Pi visible camera and surgeon’s eye-surrogate camera. For each design a projective transform was determined at 50 cm to align the FAR-Pi image and eye-surrogate camera images. This transform was then applied to FAR-Pi images obtained at 40–60 cm, and the vertical and horizontal alignment error between transformed images and eye-surrogate camera images was determined by comparing the position of the resolution target group 1.0 line pairs.

### Combining FAR-Pi components with augmented reality heads up display

The FAR-Pi system has a single white light and NIR stream, and when this non-stereoscopic stream is input into a commercial HUD, identical images are displayed to each eye. System calibration ensures alignment between the imaging module and one eye, and thus the displayed image will be aligned for one eye but misaligned for the other. This creates a perception of double vision, and to overcome this limitation we disconnected one of the eye displays of the Rokid Air AR glasses (Figure S15A-D). Additional details regarding Rokid Air modifications and coupling to FAR-Pi components are provided in Section 4 of the Supplement (Figures S16, S17, S18). Supplemental Tables S1-S4 summarize components and costs for multiple FAR-Pi implementations different choices for illumination, imaging, and computational modules.

### Statistical Analysis

Descriptive statistics were used. For the in-vivo studies, the peak LS301 SBR values for each mouse were compared between each of the 4 imaging modules with one way analysis of variance (ANOVA), using the *anova1* and *multcompare* functions in MATLAB.

## Data Availability

All data are available in the main text or the supplementary materials. Data and related code are available upon reasonable request.
